# Development and validation of a dementia risk prediction model for low- and middle-income countries: the 10/66 study

**DOI:** 10.1093/aje/kwag116

**Published:** 2026-06-02

**Authors:** Eduwin Pakpahan, Zhongyang Guan, Mario Siervo, Graciela Muniz-Terrera, Devi Mohan, Daisy Acosta, Ana Luisa Sosa, Isaac Acosta, Juan J Llibre-Rodriguez, Jorge J Llibre-Guerra, Martin Prince, Alice Worrall, Aliya Naheed, Ashleigh S Vella, Jiyang Jiang, Darren M Lipnicki, Perminder S Sachdev, Louise Robinson, Matthew Prina, Blossom C M Stephan

**Affiliations:** Applied Statistics & Data Science Research Group, School of Engineering, Physics, and Mathematics, Northumbria University, College Street, Newcastle upon Tyne NE1 8ST, United Kingdom; School of Population Health, Faculty of Health Science, Curtin University, Kent St, Bentley, Western Australia 6102, Australia; Dementia Centre of Excellence, Curtin EnAble Institute, Faculty of Health Science, Curtin University, Kent St, Bentley, Western Australia 6102, Australia; School of Population Health, Faculty of Health Science, Curtin University, Kent St, Bentley, Western Australia 6102, Australia; Dementia Centre of Excellence, Curtin EnAble Institute, Faculty of Health Science, Curtin University, Kent St, Bentley, Western Australia 6102, Australia; Heritage College of Osteopathic Medicine, Ohio University, 191 W Union St, Athens, OH 45701, United States; School of Public Health, The University of Queensland, Brisbane, 266 Herston Rd, Herston 4006, Queensland, Australia; Internal Medicine Department, Geriatric Section, Universidad Nacional Pedro Henríquez Ureña (UNPHU), 1/2, Av. John F. Kennedy 7, Santo Domingo 10203, Dominican Republic; Laboratory of the Dementias, Instituto Nacional de Neurologia y Neurocirugia “Manuel Velasco Suarez”, Av. Insurgentes Sur 3877, La Fama, Tlalpan, 14269 Ciudad de México, CDMX, Mexico; Laboratory of the Dementias, National Institute of Neurology and Neurosurgery, Av. Insurgentes Sur 3877, La Fama, Tlalpan, 14269 Ciudad de México, Mexico; Dementia Research Unit, Medical University of Havana, Ave 31 y 146. Cubanacan, CP 10600 Playa, Cuba; Department of Neurology, Washington University School of Medicine in St. Louis, 660 S. Euclid Ave., St. Louis, MO 63110-1010, United States; Department of Public Health Sciences, Faculty of Life Sciences and Medicine, King’s College London, Strand, London WC2R 2LS, United Kingdom; Institute of Mental Health, University of Nottingham, Innovation Park, Triumph Road, Nottingham NG7 2TU, United Kingdom; Non Communicable Diseases, Nutrition Research Division, icddr,b, 68, Shaheed Tajuddin Ahmed Sarani, Mohakhali, Dhaka 1212, Bangladesh; Centre for Healthy Brain Ageing (CHeBA), Discipline of Psychiatry and Mental Health, School of Clinical Medicine, Faculty of Medicine and Health, University of New South Wales, Cnr High St & Botany St, UNSW Sydney, Kensington, New South Wales 2033, Australia; Centre for Healthy Brain Ageing (CHeBA), Discipline of Psychiatry and Mental Health, School of Clinical Medicine, Faculty of Medicine and Health, University of New South Wales, Cnr High St & Botany St, UNSW Sydney, Kensington, New South Wales 2033, Australia; Centre for Healthy Brain Ageing (CHeBA), Discipline of Psychiatry and Mental Health, School of Clinical Medicine, Faculty of Medicine and Health, University of New South Wales, Cnr High St & Botany St, UNSW Sydney, Kensington, New South Wales 2033, Australia; Centre for Healthy Brain Ageing (CHeBA), Discipline of Psychiatry and Mental Health, School of Clinical Medicine, Faculty of Medicine and Health, University of New South Wales, Cnr High St & Botany St, UNSW Sydney, Kensington, New South Wales 2033, Australia; Population Health Sciences Institute, Faculty of Medical Sciences, Newcastle University, Campus for Ageing and Vitality, Newcastle upon Tyne NE4 5PL, United Kingdom; Population Health Sciences Institute, Faculty of Medical Sciences, Newcastle University, Campus for Ageing and Vitality, Newcastle upon Tyne NE4 5PL, United Kingdom; Dementia Centre of Excellence, Curtin EnAble Institute, Faculty of Health Science, Curtin University, Kent St, Bentley, Western Australia 6102, Australia

**Keywords:** dementia, Alzheimer’s disease, risk prediction, low-and middle-income countries

## Abstract

Most people with dementia live in low- and middle-income countries (LMICs), underscoring the need for LMIC-specific identification of high-risk individuals. This study aimed to develop and validate a simple dementia risk prediction model for these settings. Data from 7 10/66 Study sites were analyzed. Over 100 candidate predictors were screened based on existing models and the 2024 Lancet Commission, including LMIC-specific variables (eg, food insecurity and household assets). Predictors were selected using LASSO and modelled with the Fine–Gray method to generate a risk score. Predictive accuracy was pooled via meta-analysis. Eleven thousand one hundred forty-three participants were included, among whom 1069 (9.6%) developed dementia during follow-up. A 5-factor risk score comprising age, social engagement, physical activity, hypertension, and difficulty in handling money was developed. The pooled c-statistic was 0.75 (95% CI, 0.72-0.78), with good calibration across sites. Decision curve analysis showed a modest net benefit, with variation across countries. It is possible to predict incident dementia with reasonable accuracy using a simple model across different LMICs. Our findings support the use of context-specific risk assessment tools to identify individuals at elevated dementia risk in LMIC settings, which may inform resource allocation for dementia care services and public health planning.

## Introduction

Dementia is a major global public health priority.^1^ While research toward a cure is ongoing, new strategies for early identification of high-risk individuals are needed to support research planning and resource allocation. This is particularly important for low- and middle-income countries (LMICs) where approximately 60% of people with dementia reside.^2^ Yet dementia is under-researched and under-funded in LMICs, resulting in significant knowledge gaps.

Numerous dementia risk prediction models have been developed; however, they have been predominantly derived from high-income country (HIC) samples.^3-6^ Models not based on Alzheimer’s disease (AD) core biomarkers typically incorporate variables previously associated with dementia, including demographic (eg, age, sex, education), health (eg, cardio-metabolic and mental health), social (eg, isolation), cognitive (eg, global and domain-specific function), functional (eg, activities of daily living and instrumental activities of daily living), genetic (apolipoprotein E4 status), and lifestyle (eg, poor diet, smoking, alcohol consumption, and physical inactivity) factors. However, most models demonstrate poor predictive accuracy (ie, c-statistic <0.70), limited external validity, and lack of generalizability. Indeed, recent work by our group found that models developed in HICs cannot be directly extrapolated to LMICs.^7^ This likely reflects fundamental differences between regions, including variations in baseline dementia incidence, risk factor prevalence, and clustering, life-course exposures (eg, education attainment and poverty), socioeconomic status, and diagnostic practices. Such contextual differences influence both the baseline hazard and the strength of predictor–outcome associations, reducing the applicability of models derived in HIC data sources when applied in LMICs.^7^ Therefore, there is an urgent need to develop LMIC-specific models that account for differences in risk factor profiles (eg, age and disease distributions), types of risk factors (eg, LMIC-specific factors such as illiteracy, food insecurity, and socioeconomic determinants), and population characteristics (eg, ethnicity and country income status).^6^

The aim of this study was to develop and externally validate an accurate model for predicting dementia risk in diverse LMIC settings. A context-specific approach to dementia risk prediction is crucial to support evidence-based resource allocation, risk stratification for clinical trials and observational studies, and planning of dementia care services in LMIC settings.

## Methods

### Data source

Data were obtained from the 10/66 Study, which includes sites in China, Cuba, Dominican Republic, India, Mexico, Peru, Puerto Rico, and Venezuela.^8^^,^^9^ All individuals aged ≥65 years and living in geographically defined catchment areas in each country were eligible for participation. Participants were recruited through a door-knocking census that avoided high-income areas. Baseline interviews took place between 2003 and 2007 in all countries except Puerto Rico (where baseline interviews were completed between 2007 and 2010). More than 80% of the target population responded, with sample sizes ranging from 1900 to 3000 across countries. Follow-up interviews took place between 2007 and 2010 (2012 and 2013 in Puerto Rico) in 7 of the original sites: China, Cuba, the Dominican Republic, Mexico, Peru, Puerto Rico, and Venezuela. Study locations included 7 urban areas (1 per country) and 3 rural sites (in China, Mexico, and Peru). Only data from these 7 countries were analyzed in this study. The current study followed reporting guidelines of the Transparent Reporting of a multivariable prediction model for Individual Prognosis Or Diagnosis (TRIPOD + AI; Appendix S1).

### Outcome

Dementia was diagnosed using the cross-culturally validated 10/66 dementia diagnosis algorithm; full details have been previously published.^10-12^ The algorithm was specifically developed to be culturally and educationally fair and incorporates cognitive test scores including the Community Screening Instrument for Dementia (CSI-D) COGSCORE and the modified Consortium to Establish a Registry for Alzheimer’s Disease (CERAD) 10-word list-learning task with delayed recall. In addition, informant reports of cognitive and functional decline from the CSI-D RELSCORE and diagnostic output from the Geriatric Mental State (GMS) examination are also utilized, enabling consistent and reliable dementia diagnoses across diverse LMIC settings.^8^

### Candidate predictors

From a list of over 100 potential candidate predictors (Appendices S2 and S3), identified from the 2024 Lancet Commission on Dementia and our previous systematic reviews^3-5^^,^^13^ (Appendix S3, Table S1), 53 were available in the 10/66 Study (Appendix S3, Table S2). Additionally, we considered context-specific variables (food insecurity, late-life residence and assets) that might increase dementia risk in LMICs and were available in the 10/66 Study (Appendix S3, Table S3).

### Model development

The 7 10/66 Study countries were randomly split into development (China, Cuba, Dominican Republic, Puerto Rico, and Venezuela) and validation (Mexico and Peru) samples. Predictor selection was performed using a 2-stage approach. First, least absolute shrinkage and selection operator (LASSO) Cox regression was employed to identify potential dementia predictors using data from the combined 5 10/66 development sites.^14^ The optimal penalty parameter was determined through 10-fold cross-validation to minimize the partial deviance. Predictors selected through LASSO were then refined using stepwise Cox regression with a mixed approach (forward selection with backward elimination), applying entry and removal criteria both set at *P* < .05. The final set of predictors was incorporated into a Fine–Gray competing risks regression model to account for death as a competing event.^15^ Duration of follow-up was defined as the time from baseline assessment to dementia diagnosis, the end of follow-up, death, or loss to follow-up, whichever came first. The Schoenfeld residual test was used to check the proportional hazards assumption, and no violations were found. Fine–Gray model coefficients from the development sample were then transformed into a risk score using established methods.^16^ To examine possible geographic heterogeneity in dementia risk, we constructed country-specific Fine–Gray models using the same set of predictors selected from the overall analysis, and country-specific risk scores were also derived. This allowed us to evaluate the applicability of a single scoring system across populations from different LMICs. Missing data for all predictors included in the final model were handled using a complete-case (case-wise deletion) approach. Sample size adequacy for model development was assessed, with full details provided in Appendix S3, Table S4.

### Model validation

Both internal (within each of the 5 development countries and the combined development sample) and external (within each of the 2 validation countries and the combined validation sample) validation of the risk score were undertaken over a 3-year time horizon. The discriminative performance of the risk score was assessed using the concordance statistic (c-statistic) with its 95% confidence interval (95% CI).^17^ Calibration at 3 years was assessed by comparing the agreement between the observed proportion of dementia cases and predicted probabilities of developing dementia derived from the risk score. The DeLong test, adapted for survival analysis, was employed to determine whether the c-statistic values from the risk score model were statistically different from those of a model incorporating age alone,^18^ thereby providing a benchmark for the incremental discriminative value of the additional predictors beyond age alone.^19^ Clinical utility was evaluated using Decision Curve Analysis (DCA). Net benefit for incident dementia at 3 years was calculated across threshold probabilities ranging from 0% to 20%. Four strategies were compared: (1) the full multivariable model, (2) an age-only model, (3) screening all individuals (“treat all”), and (4) screening no individuals (“treat none”).^20^

### Meta-analysis

The pooled estimate of the predictive accuracy of the model was calculated using a random-effects meta-analysis: (1) across all 5 10/66 development countries, (2) across the 2 validation countries, and (3) across all 7 10/66 countries combined. Heterogeneity was assessed using the *I*^2^ statistic, with *I*^2^ values of 75%-100% indicative of substantial heterogeneity.

### Sensitivity analysis

To assess the robustness of the findings, 3 sensitivity analyses were conducted. First, the original model was extended through the inclusion of 3 LMIC-specific predictors (food insecurity, late-life residence, and household assets), and its discriminative performance was compared with that of the original model across all 7 10/66 countries individually and combined. Second, sex-stratified analyses were performed to assess whether the risk score demonstrated consistent discriminative performance between males and females across all countries. The third sensitivity analysis examined model performance among Latin American countries by excluding China from the development sample. The development sample thus comprised 4 Latin American countries (Cuba, Dominican Republic, Puerto Rico, and Venezuela), while Mexico and Peru remained as the validation sample. This allowed for evaluation of whether the inclusion of a non-Latin American country (ie, China) influenced the model’s performance and generalizability within the Latin American context. In all analyses, differences in discrimination (ie, c-statistic values) were compared using the DeLong test. All statistical analyses were performed using Stata 18 and R 4.4.1.

## Results

At baseline, 15 016 participants were recruited; among these, 1429 with prevalent dementia and 2444 who were lost to follow-up were excluded. Consequently, 11 143 participants across the 7 10/66 Study countries were included in this analysis, among whom 1069 (9.6%) developed dementia during follow-up. The mean age ranged from 71.8 to 75.4 years across sites, and most participants were women (62.6%). Mean follow-up across all sites combined was 3.8 years (Table 1).

**Table 1:** 10/66 Study sample characteristics.

	**Total sample** ***n*/*N* (%) or mean (SD)**	**China** ***n*/*N* (%) or mean (SD)**	**Cuba** ***n*/*N* (%) or mean (SD)**	**DR** ***n*/*N* (%) or mean (SD)**	**Mexico** ***n*/*N* (%) or mean (SD)**	**Peru** **n/N (%) or mean (SD)**	**Puerto Rico** ***n*/*N* (%) or mean (SD)**	**Venezuela** ***n*/*N* (%) or mean (SD)**	***P*-value** ^**a**^
Follow-up, years	3.8 (1.3)	4.4 (1.2)	4.0 (1.3)	4.3 (1.5)	2.8 (0.6)	3.0 (0.8)	4.0 (1.2)	3.9 (1.1)	<.0001
Dementia at follow-up	1069/11143 (9.6%)	207/1832 (11.3%)	182/2300 (7.9%)	165/1441 (11.5%)	130/1521 (8.6%)	77/1323 (5.8%)	153/1373 (11.1%)	155/1353 (11.5%)	<.0001
Female	6973/11137 (62.6%)	1019/1832 (55.6%)	1480/2300 (64.4%)	948/1439 (65.9%)	963/1521 (63.3%)	791/1323 (59.8%)	923/1369 (67.4%)	849/1353 (62.8%)	<.0001
Baseline age, years	73.8 (6.6)	72.8 (5.9)	74.4 (6.6)	74.6 (7.2)	73.6 (6.2)	74.2 (7.0)	75.4 (6.5)	71.8 (6.4)	<.0001
Education									<.0001
Secondary or higher	3408/11111 (30.7%)	409/1832 (22.3%)	993/2295 (43.4%)	150/1435 (10.5%)	186/1519 (12.2%)	564/1313 (43.0%)	836/1373 (60.9%)	270/1344 (20.1%)	
Primary	3231/11111 (29.1%)	472/1832 (25.8%)	771/2295 (33.6%)	266/1435 (18.5%)	286/1519 (18.8%)	493/1313 (37.6%)	260/1373 (18.9%)	683/1344 (50.8%)	
Not completed primary	4468/11111 (40.2%)	951/1832 (51.9%)	531/2295 (23.1%)	1019/1435 (71.0%)	1047/1519 (68.9%)	256/1313 (19.5%)	273/1369 (19.9%)	391/1344 (29.1%)	

Abbreviations: DR, Dominican Republic; SD, standard deviation.

a Between-country heterogeneity in sample characteristics was assessed using analysis of variance (ANOVA) for continuous variables and chi-square tests for categorical variables.

### Model development and validation

Using LASSO followed by stepwise Cox regression, 5 predictors were selected from 53 candidate variables using the combined 5-country development dataset: age, social engagement, physical activity, hypertension, and difficulty in handling money. The sample size calculation confirmed that our dataset far exceeded the minimum number of participants and events required for stable model development (Appendix S3, Table S4). The pattern of missing data for each variable across the different 10/66 Study countries ranged from none to 19% (*n* = 257 for hypertension in Venezuela) (Appendix S3, Table S5). The Fine–Gray analysis results for the combined development sample and each 10/66 Study site separately are presented in Table 2. The results demonstrated variability in the pattern of associations between the predictor variables and dementia. Age was the only variable that consistently predicted dementia across all sites (sub-distribution hazard ratio [SHR] range: 1.06-1.11). Physical inactivity demonstrated particularly strong associations in Puerto Rico (SHR = 4.01) and Venezuela (SHR = 2.92). Furthermore, difficulty in handling money demonstrates variation across countries, with notably strong effects in China (SHR = 4.18) and Venezuela (SHR = 2.89).

**Table 2:** Fine–Gray model results in the development sample combined and each 10/66 study country.

**Predictors**	**Development sample**	**China**	**Cuba**	**DR**	**Mexico**	**Peru**	**Puerto Rico**	**Venezuela**
	**SHR (95% CI)**	**SHR (95% CI)**	**SHR (95% CI)**	**SHR (95% CI)**	**SHR (95% CI)**	**SHR (95% CI)**	**SHR (95% CI)**	**SHR (95% CI)**
Age (continuous)	**1.07 (1.06, 1.08)**	**1.10 (1.07, 1.12)**	**1.06 (1.04, 1.08)**	**1.06 (1.04, 1.08)**	**1.09 (1.06, 1.11)**	**1.11 (1.08, 1.14)**	**1.08 (1.05, 1.11)**	**1.10 (1.07, 1.12)**
Social engagement								
High	Ref.	Ref.	Ref.	Ref.	Ref.	Ref.	Ref.	Ref.
Medium	1.17 (0.93, 1.48)	1.22 (0.51, 2.90)	1.46 (0.80, 2.64)	1.03 (0.65, 1.65)	0.83 (0.49, 1.38)	1.39 (0.74, 2.60)	1.33 (0.84, 2.08)	1.39 (0.84, 2.29)
Low	**1.26 (1.01, 1.58)**	1.25 (0.54, 2.89)	**2.40 (1.36, 4.24)**	1.13 (0.71, 1.80)	0.97 (0.60, 1.56)	**2.00 (1.08, 3.69)**	0.80 (0.51, 1.25)	1.07 (0.64, 1.78)
Physical activity								
Very	Ref.	Ref.	Ref.	Ref.	Ref.	Ref.	Ref.	Ref.
Fairly active	**1.28 (1.03, 1.58)**	1.33 (0.83, 2.13)	1.44 (0.94, 2.23)	0.96 (0.64, 1.43)	1.03 (0.62, 1.73)	1.11 (0.58, 2.14)	**2.11 (1.01, 4.42)**	1.78 (0.87, 3.62)
Not (very)	**1.76 (1.43, 2.16)**	1.52 (0.98, 2.37)	1.51 (0.95, 2.40)	1.13 (0.78, 1.66)	1.50 (0.88, 2.54)	**1.92 (1.00, 3.68)**	**4.01 (1.88, 8.53)**	**2.92 (1.46, 5.84)**
Hypertension								
No	Ref.	Ref.	Ref.	Ref.	Ref.	Ref.	Ref.	Ref.
Yes	1.12 (0.97, 1.29)	1.25 (0.94, 1.65)	1.34 (0.98, 1.84)	1.26 (0.92, 1.73)	0.88 (0.61, 1.26)	0.34 (0.12, 0.93)	1.18 (0.82, 1.68)	0.86 (0.60, 1.23)
Difficulty in handling money								
No	Ref.	Ref.	Ref.	Ref.	Ref.	Ref.	Ref.	Ref.
Yes	**2.11 (1.59, 2.79)**	**4.18 (2.13, 8.21)**	**1.87 (1.04, 3.39)**	1.40 (0.81, 2.43)	1.87 (0.94, 3.73)	1.07 (0.33, 3.49)	**2.57 (1.19, 5.52)**	**2.89 (1.63, 5.13)**

Abbreviations: CI, confidence interval; DR, Dominican Republic; SHR, sub-distribution hazard ratio.

A point-based risk score (ranging from 0 to 9) was derived using the coefficients from the Fine–Gray analysis of the combined development sample (see Figure 1 and Appendix S3, Table S6 for the points allocated to each variable; see Figure 1 for the associated probabilities of progressing to dementia for each score). The distribution of total risk scores in the combined development sample, combined validation sample, and each individual 10/66 Study country is shown in Appendix S3, Figure S1. The distribution of total risk scores showed similar patterns across countries, predominantly ranging from 0 to 7. Country-specific risk scores derived from the Fine–Gray analysis of each country are presented in Appendix S3, Table S7. The discriminative performance of the risk score is shown in Table 3 and Figure 2. In the combined development sample, the c-statistic was 0.74 (95% CI, 0.72-0.76) overall, with values ranging from 0.71 (95% CI, 0.67-0.75) in the Dominican Republic to 0.78 (95% CI, 0.73-0.83) in Venezuela. The discriminative accuracy of the risk score was significantly superior to age alone (0.70; 95% CI, 0.68-0.72) in the 5 development countries combined (*P* < .001).

**Figure 1:**
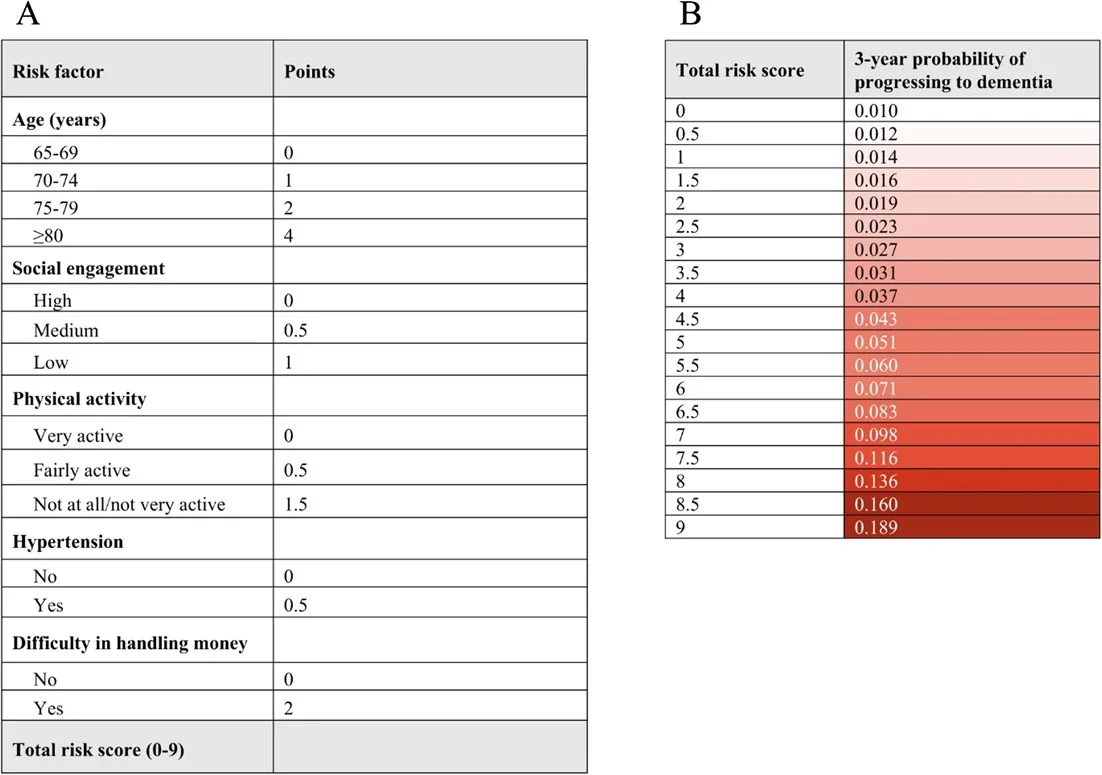
Point-based dementia risk score and corresponding 3-year predicted probabilities. Legend: Panel (A) presents the point-based scoring system derived from the Fine–Gray model, consisting of 5 predictors: age, social engagement, physical activity, hypertension, and difficulty handling money. Panel (B) displays the predicted 3-year probability of progressing to dementia corresponding to each possible total risk score.

**Figure 2:**
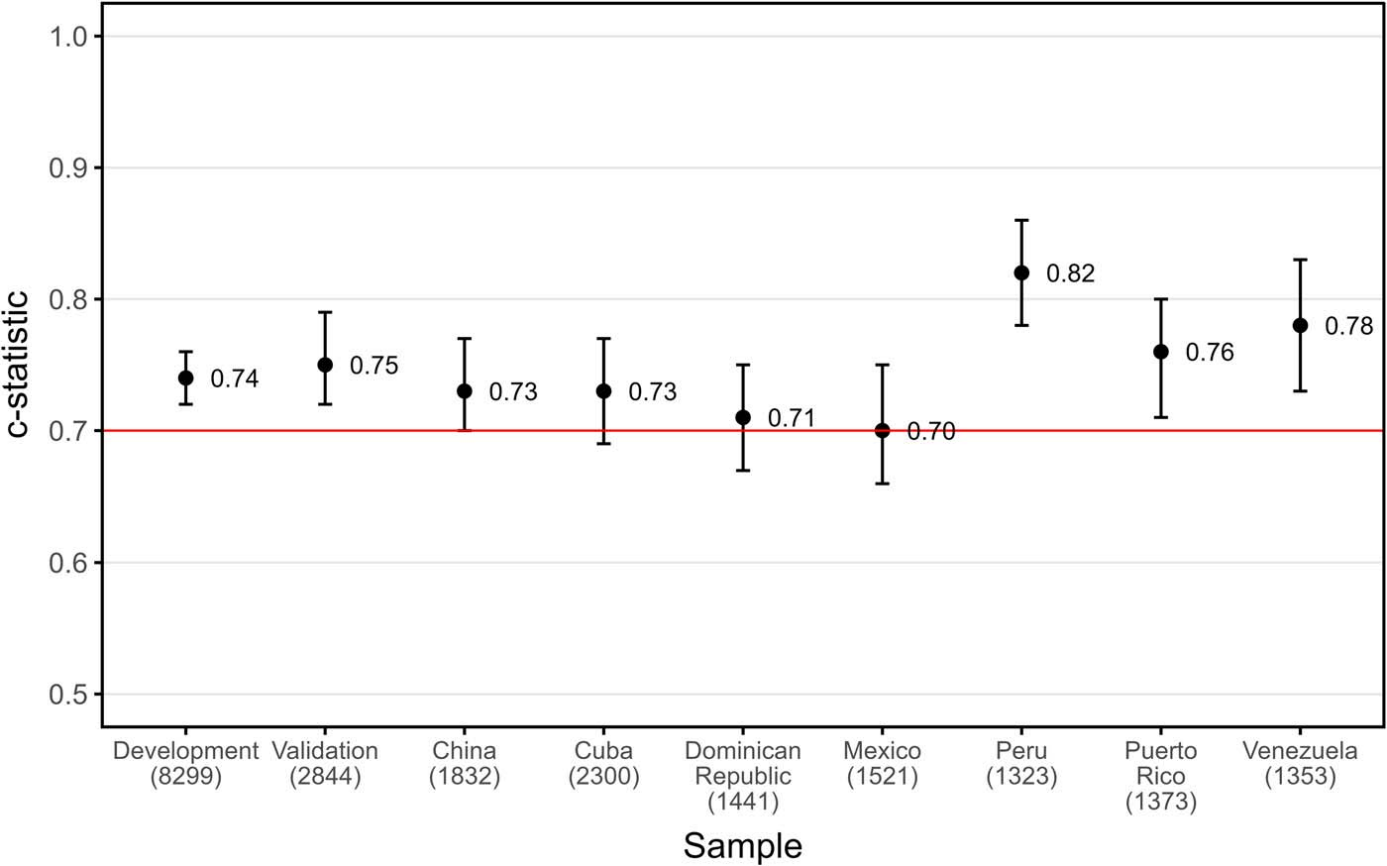
Comparison of the discrimination performance (c-statistic) of the risk score-based Fine-Gray model at 3 years. The horizontal reference line indicates the cut-off value for acceptable predictive accuracy (c-statistic ≥0.70).

**Table 3:** Discrimination and calibration performance of the risk score-based Fine–Gray model.

**Sample**		**Discrimination**			**Calibration**	
	**Follow-up, years Mean (SD)**	**Risk score C-statistic (95% CI)**	**Age alone C-statistic (95% CI)**	**Comparison (*P*-value)**	**Intercept (95% CI)**	**Slope (95% CI)**
All countries (overall)	3.8 (1.3)	0.74 (0.72, 0.76)	0.71 (0.69, 0.72)	<.001	0.007 (−0.003, 0.016)	0.956 (0.843, 1.069)
Development sample (overall)	4.1 (1.3)	0.74 (0.72, 0.76)	0.70 (0.68, 0.72)	<.001	0.007 (−0.005, 0.019)	0.974 (0.843, 1.106)
Validation sample (overall)	2.9 (0.7)	0.75 (0.72, 0.79)	0.73 (0.69, 0.76)	.006	0.007 (−0.008, 0.021)	0.904 (0.679, 1.129)
China	4.4 (1.2)	0.73 (0.70, 0.77)	0.72 (0.68, 0.76)	.123	0.002 (−0.024, 0.028)	0.985 (0.740, 1.230)
Cuba	4.0 (1.3)	0.73 (0.69, 0.77)	0.70 (0.66, 0.74)	.002	0.007 (−0.013, 0.027)	1.065 (0.739, 1.392)
DR	4.3 (1.5)	0.71 (0.67, 0.75)	0.69 (0.65, 0.73)	.104	0.009 (−0.037, 0.062)	0.961 (0.609, 1.313)
Mexico	2.8 (0.6)	0.70 (0.66, 0.75)	0.69 (0.65, 0.74)	.360	0.004 (−0.022, 0.031)	0.950 (0.608, 1.292)
Peru	3.0 (0.8)	0.82 (0.78, 0.86)	0.79 (0.74, 0.84)	.044	0.010 (−0.005, 0.025)	0.826 (0.508, 1.143)
Puerto Rico	4.0 (1.2)	0.76 (0.71, 0.80)	0.72 (0.67, 0.76)	.001	0.008 (−0.018, 0.034)	0.975 (0.708, 1.242)
Venezuela	3.9 (1.1)	0.78 (0.73, 0.83)	0.72 (0.68, 0.77)	.001	0.007 (−0.021, 0.035)	0.946 (0.672, 1.220)

Abbreviations: CI, confidence interval; DR, Dominican Republic.

In the validation sample, the c-statistic of the risk score was 0.75 (95% CI, 0.72-0.79) overall, ranging from 0.70 (95% CI, 0.66-0.75) in Mexico to 0.82 (95% CI, 0.78-0.86) in Peru. The discriminative performance of the risk score was significantly superior to age alone (0.73; 95% CI, 0.69-0.76) in the 2 validation countries combined (*P* = .006).

Regarding calibration, the risk score was well calibrated in the 5 development countries combined (Appendix S3, Figure S2). Country-specific calibration intercepts and slopes were also close to ideal values (Table 3), indicating generally good agreement between predicted and observed 3-year risks. Mild deviations were noted in some settings, that is, slight underprediction at higher predicted risks in Peru, but these differences were modest in magnitude.

DCA results are presented in Appendix S3, Figure S3. Across the overall 10/66 Study sample and within each country, the full model yielded higher net benefit than the “treat all” and “treat none” strategies across most of the 0%-20% threshold range. The difference between the multivariable and age-only models was modest in most sites.

### Pooled predictive accuracy

Meta-analysis results demonstrated that the pooled *C* statistics across all 7 countries (0.75; 95% CI, 0.72-0.78), the 5 development countries (0.74; 95% CI: 0.72-0.76), and the 2 validation countries (0.76; 95% CI, 0.64-0.88) were similar and high (Figure 3). Overall heterogeneity across all countries was, however, high (*I*^2^ = 75%). Subgroup analyses revealed greater heterogeneity across the 2 validation countries (*I*^2^ = 93%) compared to the 5 development countries (*I*^2^ = 34%).

**Figure 3:**
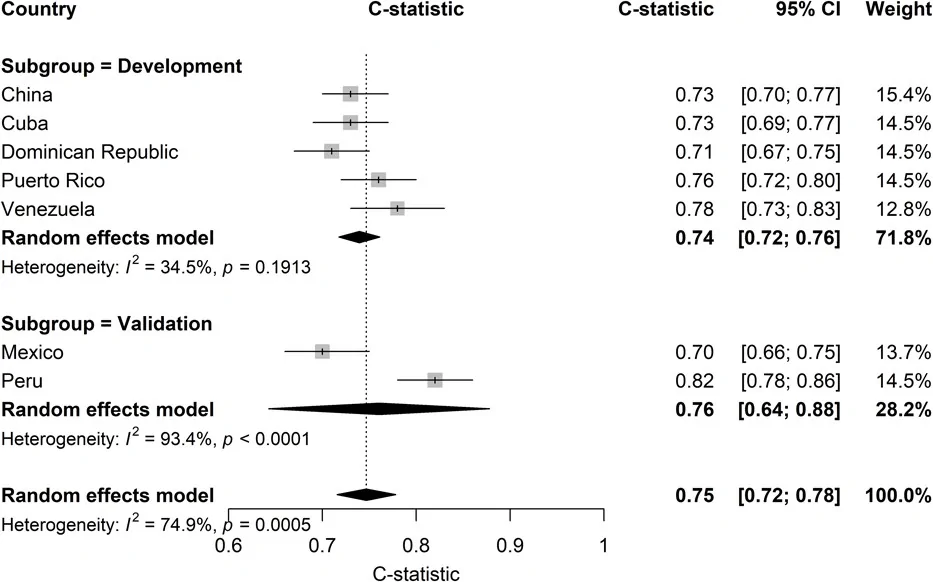
Pooled predictive accuracy of the risk score. Legend: Random-effects meta-analysis results showing the pooled predictive accuracies of the risk score–based Fine–Gray model.

### Sensitivity analyses

The extended risk score, incorporating 3 LMIC-specific predictors, showed comparable c-statistic values to the 5-factor model (all *P* > .05; Tables S8 and S9). Further, the discriminative performance of the risk score did not differ significantly between males and females (Appendix S3, Table S10). The sensitivity analysis excluding China also demonstrated similar results (Appendix S3, Tables S11 and S12). Discriminative performance of the risk score was significantly superior to the age alone model in the 4 development countries combined, the 2 validation countries combined, and all 6 countries combined (Appendix S3, Table S12).

## Discussion

In this study, we developed a simple risk score for predicting dementia in LMICs, incorporating 5 predictors spanning multiple domains: demographic (age), social engagement, vascular health (hypertension), lifestyle (physical inactivity), and functional capacity (difficulty in handling money) variables. The model demonstrated good to excellent accuracy for predicting incident dementia across 7 diverse LMICs, including sites in East Asia (China), the Caribbean (Cuba, Dominican Republic, Puerto Rico), South America (Peru, Venezuela), and North America (Mexico). These findings provide a framework for identifying individuals at elevated dementia risk in LMICs to assist resource prioritization and planning.

The 5 predictors selected in our model (age, social engagement, hypertension, physical activity, and difficulty in handling money) have been previously shown to be associated with dementia in epidemiology studies.^21^^,^^22^ While our study focuses on the predictive utility within a risk stratification framework rather than establishing causal relationships, it is worth noting that these factors align with findings from prior research.^23^ Interestingly, some risk factors identified in the 2024 Lancet Commission on Dementia,^13^ including low education, diabetes, and depression, were not selected by our data driven approach. This suggests either differential predictive strength of these risk factors across the 10/66 Study sites or potential methodological considerations related to measurement and population characteristics. Further, LMIC-specific variables such as household assets, food insecurity, and late-life residence did not enhance predictive accuracy when added to the model. This may suggest that the core predictive factors for dementia may be relatively consistent across socioeconomic contexts, though further work is needed to explore additional contextual variables that may be salient in LMIC settings, including war exposure, famine, and adverse childhood experiences.^6^^,^^24^

The overall performance of the risk score was encouraging across the 7 10/66 Study countries. Discriminative ability was good when combined across all 7 sites (pooled c-statistic = 0.75; 95% CI, 0.72-0.78), comparable to existing models and superior to HIC-based models validated in the same sample (c-statistic range: 0.55-0.72).^3^^,^^7^ However, some variability in discrimination was observed across individual countries. Indeed, in Mexico, the c-statistic of the risk score was at the 0.70 threshold. Differences in discrimination across countries likely reflect variation in underlying risk factor distributions, socioeconomic conditions, and the prevalence and severity of dementia at follow-up. Variation in sample sizes and the number of incident dementia cases may also influence the stability and precision of site-specific estimates.

The risk score demonstrated good overall calibration in the combined development sample, with predicted and observed 3-year risks showing close agreement. Country-specific assessments also indicated generally acceptable calibration, with only mild deviations in a few settings. These deviations were modest in magnitude and are expected given differences in population characteristics and event distributions across countries. Overall, the results suggest that the model is broadly well calibrated in diverse LMIC populations. Nevertheless, as done in the cardiovascular field, simple country-level recalibration procedures (eg, adjusting the intercept or slope) may further improve accuracy in settings where local risk distributions differ from those of the pooled development dataset.^6^^,^^25^

The Decision Curve Analysis revealed that the multivariable model offers limited incremental clinical utility over an age-only model in most settings. With the exception of Cuba, where the full model demonstrated clear gains in net benefit, the additional predictors provided negligible improvement beyond age alone. This raises important questions about the practical value of adding complexity beyond a simple age-based approach.

We acknowledge that our model does not demonstrate sufficient incremental benefit to justify its use for population-level screening or clinical decision-making in most LMIC settings. For such applications, age alone would likely be equally effective and far more practical. The model may retain value for specific research purposes—such as enriching clinical trial enrolment—but simpler age-based approaches may be preferable for most practical applications.

It is important to note that across the 10/66 Study countries, there was variability in the associations between the predictor variables and incident dementia, with age being the only variable to demonstrate a consistently significant association across all countries. Physical inactivity showed particularly strong associations in Puerto Rico and Venezuela, while difficulty in handling money demonstrated stronger effects in China and Venezuela. These cross-country differences may arise from a combination of true contextual variation and differences in measurement quality. For example, variations in education, lifestyle patterns, and cardiometabolic health may influence how strongly certain variables predict dementia risk, while differences in diagnostic resources, reporting accuracy, and cultural perceptions of psychosocial factors may lead to differential measurement error across sites. Importantly, even when variables are operationalized using identical instruments, their cultural meaning, functional implications, and reporting norms may differ across countries, resulting in measurement non-equivalence. Such differences can contribute to the observed heterogeneity in predictor associations and influence the generalizability of individual predictors across LMIC settings. From a risk prediction perspective, it remains important to determine the most salient risk factors within specific cultural and geographical contexts to improve model transportability and inform future recalibration efforts.

### Strengths and limitations

The study has several key strengths. First, model development was undertaken using the 10/66 Study, which features a large sample size, consistent assessment protocols across sites, and comprehensive information on dementia risk factors. Second, access to multiple LMIC sites enhances the generalizability of the results. Indeed, these countries differ in geography, economy, political systems, healthcare infrastructure, ethnic diversity, and cultural contexts. Third, risk factors were selected based on previous reviews and LMIC-specific research, ensuring the model incorporated key dementia predictors in LMIC settings. Fourth, we selected exclusively predictors that are simple to obtain. Simplicity in calculating risk is essential in LMIC settings due to limited resources, accessibility concerns, and the need to accommodate cultural and linguistic diversity. Fifth, successful external validation across multiple LMICs enhances credibility and potential to inform policy regarding dementia risk identification in real-world healthcare settings.

However, several limitations should be acknowledged. First, a proportion of participants did not complete a follow-up cognitive assessment and were therefore excluded from the analytic cohort. Importantly, this pattern was primarily driven by structural differences in the 10/66 Study design rather than participant attrition. The India site conducted only mortality follow-up without repeat cognitive assessments, so all Indian participants were excluded from incidence-based analyses. Among the remaining sites, loss to cognitive follow-up was modest; however, individuals without follow-up may differ systematically from those retained.^8^ Such selective loss to follow-up may lead to underestimation of dementia incidence and attenuation of predictive performance, particularly calibration, if those without follow-up were at higher underlying risk. Second, the accuracy of self-reported variables (such as smoking, diabetes, and education) might be limited by memory, cross-cultural understanding, or social desirability bias. Third, the model exhibited heterogeneity in pooled c-statistic estimates, with greater heterogeneity in the validation sample, potentially reflecting differences in healthcare systems and risk factor distributions across these diverse settings. Fourth, the model was developed and validated to predict all-cause dementia; whether different models are needed to predict dementia subtypes (eg, Alzheimer’s disease and vascular dementia) requires further investigation. Fifth, the model incorporates only late-life health risk factor data. Cardio-metabolic health and lifestyle factors have been previously associated with an increased risk of dementia in HICs when assessed in midlife, with no association or reverse associations sometimes observed when assessed in later life.^26^^,^^27^ However, longitudinal mid-to-late life health and dementia data are rare, even more so in LMICs. Reliance on late-life measurements may attenuate or modify observed associations due to treatment effects, age-related physiological changes, and survivorship bias. Sixth, the average follow-up of 3.8 years provides important early insights but may not fully capture longer-term cognitive trajectories; however, our competing-risk framework appropriately accounts for death as a competing event. Seventh, in the current study, the dementia cases were identified based on the 10/66 dementia diagnosis algorithm, which does not closely correspond with the widely used Diagnostic and Statistical Manual of Mental Disorders (DSM-IV) diagnostic criteria. The DSM-IV criteria identify more severe dementia, whereas the 10/66 algorithm captures a broader spectrum of dementia cases. Although the 10/66 algorithm has demonstrated high sensitivity and specificity in validation studies,^10^^,^^11^^,^^28^^,^^29^ some diagnostic uncertainty is unavoidable. This residual misclassification—concentrated mainly among borderline cases—may modestly attenuate the achievable discrimination of the model. Eighth, the relatively small number of studies in the meta-analysis may limit generalization to the broader population. Ninth, the Decision Curve Analysis demonstrated that the multivariable model offered minimal improvement over an age-only model in most countries, with only Cuba showing substantial gains. This suggests that age alone may be sufficient for most practical applications and that the full model is justified primarily for specific research purposes rather than routine clinical use or population-based screening. Tenth, the predictor selection strategy, which combined LASSO and stepwise regression, may preferentially retain variables with the strongest statistical signals while excluding conceptually important predictors that are correlated with stronger variables. The omission of factors such as diabetes may reflect statistical competition rather than lack of predictive relevance. Notably, sensitivity analyses conducted as part of the original modelling plan, including models with and without LMIC-specific predictors, produced comparable discrimination. These findings support the robustness of the final 5-factor model despite the data-driven nature of the selection process.

Finally, the study population was drawn from geographically defined catchment areas rather than nationally representative samples. Although these areas captured diverse socioeconomic and cultural settings, they may not fully reflect national-level heterogeneity, which should be considered when interpreting the generalizability of the risk score beyond the catchment populations.^8^

## Conclusions

Accurate identification of individuals at elevated dementia risk is essential for efficient allocation of healthcare resources and research planning in LMICs, where the burden of dementia is increasing significantly. We present a parsimonious and clinically feasible dementia risk prediction model that demonstrated good discrimination and calibration across 7 diverse LMIC settings. The model has potential to support identification of high-risk individuals for observational studies and clinical trials, inform risk stratification for research enrollment, and guide evidence-based planning of dementia care services in resource-limited settings. Further validation across additional LMIC regions, including South Asia, the Middle East and North Africa, Sub-Saharan Africa, South America, and Europe, is warranted to establish the model’s transportability. Future work should evaluate whether the factors identified in our model represent causal risk factors through intervention studies, including randomized controlled trials.

## Use of artificial intelligence (AI) tools

No AI tools were used for this work.

## Supplementary Material

Web_Material_kwag116

## Data Availability

The data used in this study are part of the 10/66 Dementia Research Group’s research and are accessible upon request. Access is granted after meeting the requirements for data usage outlined by the 10/66 Dementia Research Group. Researchers can access the data through the ADDI platform at https://fair.addi.ad-datainitiative.org/#/data/collections/1066-dementia-research-group or GAAIN at https://www.gaaindata.org/partner/1066. All study questionnaires are available online (https://www.alz.co.uk/1066/).
