# Supplementary material for: Development and validation of a dementia risk prediction model for low- and middle-income countries: the 10/66 study
Source: Am J Epidemiol. 2026 Jun 2;195(11):2710–8. doi: 10.1093/aje/kwag116 (PMC13733507; doi:10.1093/aje/kwag116)
Supplement: Supplementary file 1 [file web_material_kwag116.zip › AJE_Appendices_S2 and S3_27052026_FINAL_V1.docx]

**Supplementary Material: Development and validation of a simple dementia risk prediction model for low- and middle-income countries: the 10/66 Study**

**CONTENTS**

**Appendix S2. Methods**	2

**Appendix S3. Tables and figures**	2

**Table S1** Full list of candidate predictors	3

**Table S2** Description of the potential predictors available in the 10/66 Study	4

**Table S3** Description of the LMIC-specific predictors available in the 10/66 Study	9

**Table S4** Sample size calculation for the survival prediction model	10

**Table S5** Missing data for the selected predictors across the 10/66 study countries	11

**Table S6** Point-based risk score (Fine-Gray model) calculations and scoring	12

**Table S7** Point-based risk score for each country	13

**Table S8** Point-based risk score (with LMIC-specific predictors) calculations and scoring	14

**Table S9** Discrimination performance of the risk score-based Fine-Gray model (with and without LMIC-specific predictors) at 3-year time horizon	15

**Table S10** Discrimination performance of the risk score-based Fine-Gray model (sex-stratified) at 3-year time horizon	16

**Table S11** Point-based risk score (excluding China) calculations and scoring	17

**Table S12** Discrimination performance of the risk score-based Fine-Gray model (excluding China) at 3-year time horizon	18

**Figure S1** Distribution of total risk score	19

**Figure S2** Calibration plot of the risk score-based Fine-Gray model	20

**Figure S3** Decision curve analysis for 3-year dementia risk prediction by country	21

**Appendix S2. Methods**

Variables for model inclusion were selected based on four criteria: (1) they had been used in models identified in previous systematic reviews [1-4]; (2) they had been used in models externally validated in LMICs, including the Cardiovascular Risk Factors, Aging and Dementia (CAIDE) risk score; the Study on Aging, Cognition and Dementia (AgeCoDe) model; the Australian National University Alzheimer's Disease Risk Index (ANU-ADRI); the Brief Dementia Screening Indicator (BDSI); and the Rotterdam Study Basic Dementia Risk Model (BDRM) [5]; (3) they were reported in the 2024 Lancet Commission on Dementia (n=14 factors in life-course order including less education (early life); hearing loss, high low-density lipoprotein (LDL) cholesterol, depression, traumatic brain injury, physical inactivity, diabetes, smoking, hypertension, obesity, and excessive alcohol consumption (midlife); as well as social isolation, air pollution, and visual loss (late life)) [6]; and (4) they were simple and inexpensive to collect—variables such as neuroimaging, genetic data, and detailed neuropsychological testing were excluded due to their limited affordability and feasibility for large-scale use in LMIC settings.

Reference:

1.	Alshahrani M, Sabatini S, Mohan D, et al. Dementia risk prediction modelling in low- and middle-income countries: current state of evidence. *Front Epidemiol* 2024;**4**:1397754.

2.	Brain J, Kafadar AH, Errington L, et al. What's New in Dementia Risk Prediction Modelling? An Updated Systematic Review. *Dement Geriatr Cogn Dis Extra* 2024;**14**:49-74.

3.	Stephan BC, Kurth T, Matthews FE, Brayne C, Dufouil C. Dementia risk prediction in the population: are screening models accurate? *Nat Rev Neurol* 2010;**6**:318-326.

4.	Tang EY, Harrison SL, Errington L, et al. Current Developments in Dementia Risk Prediction Modelling: An Updated Systematic Review. *PLoS One* 2015;**10**:e0136181.

5.	Stephan BCM, Pakpahan E, Siervo M, et al. Prediction of dementia risk in low-income and middle-income countries (the 10/66 Study): an independent external validation of existing models. *Lancet Glob Health* 2020; **8**:e524-e535.

6.	Livingston G, Huntley J, Liu KY, et al. Dementia prevention, intervention, and care: 2024 report of the Lancet standing Commission. *Lancet* 2024;**404**:572-628.

**Appendix S3. Tables and Figures**

**Table S1** Full list of potential candidate predictors

| **Categories** | **Predictors** |
| --- | --- |
| Socio-demographic | Age, sex, education, social engagement, household assets, food insecurity, occupation, material deprivation, household occupancy, marital status, ethnicity, immigrant status, multilingualism, local area deprivation, residence |
| Cardio-metabolic | Obesity, diabetes, stroke, hypertension, SBP, atrial fibrillation, internal carotid artery thickness, coronary bypass surgery, congestive heart failure, cerebrovascular disease, microvascular disease, diabetic foot, acute metabolic event, heart disease, myocardial infarction, CVD, ASCVD, TIA |
| Experimental | Plasma lipid profile (e.g., lipid ratio, total Cholesterol, HDL-C, LDL-C, triglyceride) LysoPC, PC, hematocrit, white blood cell count, platelet count, mean corpuscular volume, mean corpuscular hemoglobin concentration, red cell distribution width, mean platelet volume, sodium, potassium, bicarbonate, calcium, glucose, creatinine |
| Mental & cognitive | SMI, verbal fluency test, delayed recall, MMSE score, need help with money, cognitive stimulation activities, DSST score, IST score, subjective memory complaints, FCSRT score, WMS Information score, WAIS-R Digit Symbol score, depression, loneliness, psychosis, psychological health score (CLHLS), stress, sense of belonging, mood disorder, bipolar disorders |
| Lifestyle | Smoking status, alcohol intake, fish intake, frequency of tooth brushing, fruit and vegetable consumption, juice consumption, potato consumption, physical activity, leisure activity (e.g., playing cards, watching TV or listening to the radio) |
| Anthropometric & physical | BMI, BMI-squared, WC, WHtR, weight loss, IADL impairment, physical frailty, slow physical performance, gait abnormality |
| Medical care | Aspirin use, anxiety/anxiolytics use, NSAID use, non-tricyclic antidepressant use, medication non-adherence, sedative-hypnotic use, outpatient visits, hospitalization, emergency department visit |
| Other (e.g., endocrine disorders, sensory impairments, genetic factors) | TBI, falls, insomnia, visual field defect, hearing problems, pesticide exposure, parental history of dementia, MRI white matter disease, MRI enlarged ventricles, chronic pulmonary disease, hypothyroidism, hyperthyroidism, renal failure, solid tumour without metastases, lymphoma, rheumatoid arthritis, fluid and electrolyte disorders, blood loss anaemia, self-rated health, COPD, epilepsy, Parkinson's disease, glaucoma, transplantation, gout, Crohn's and ulcerative disease, pain, inflammation, APOE status, genetic risk score (including various SNPs) |

Abbreviations: APOE, apolipoprotein E; ASCVD, atherosclerotic cardiovascular disease; BMI, body mass index; CLHLS, Chinese Longitudinal Healthy Longevity Survey; COPD, chronic obstructive pulmonary disease; CVD, cardiovascular disease; DSST, Digit Symbol Substitution Test; FCSRT, Free and Cued Selective Reminding Test; HDL-C, high-density lipoprotein cholesterol; IADL, instrumental activities of daily living; IST, Isaacs Set Test; LDL-C, low-density lipoprotein cholesterol; LysoPC, lysophosphatidylcholine; MMSE, Mini-Mental State Examination; MRI, magnetic resonance imaging; NSAID, non-steroidal anti-inflammatory drug; PC, phosphatidylcholine; SBP, systolic blood pressure; SMI, subjective memory impairment; SNPs, single-nucleotide polymorphisms; TBI, traumatic brain injury; TIA, transient ischemic attack; WAIS-R, Wechsler Adult Intelligence Scale–Revised; WC, waist circumference; WHtR, waist-to-height ratio; WMS, Wechsler Memory Scale.

**Table S2** Description of the potential candidate predictors available in the 10/66 Study

| **Candidate predictors** | **Description in 10/66 study** | **Categories** |
| --- | --- | --- |
| Age | Information obtained from participant, informant and if available from documentation. If large discrepancy existed between sources, an events calendar was used to estimate age. | NA (continuous) |
| Gender | Information obtained from the participant. | (1) Male; (2) female |
| Education | Question asked to participant: ‘How much schooling have you had?’ Options: (1) none; (2) some but did not complete primary school; (3) completed primary school; (4) completed secondary school (metric); or (5) completed tertiary school (college)/further education. | (1) Secondary/tertiary school (college)/further education; (2) Primary; or (3) not completed primary |
| Marital status | Question asked to participant: 'Are you currently married?' Options: (1) never married; (2) married/co-habiting; (3) widowed; or (4) divorced/separated. | (1) Married/ co-habiting; (2) never married/ widowed/divorced/separated |
| Occupation | Question asked to participant with other member of the household present: ‘What kind of work did you do in this job?’ Options: (1) manager/administrator (2) professional (e.g., health, teaching, legal, financial); (3) associate professional (e.g., technical, nursing, artistic); (4) clerical worker /secretary; (5) shop keeper; (6) skilled labourer (e.g., building, electrical etc.); (7) semi-skilled labourers (e.g., helper of skilled labourer); (8) unskilled labourer; or (9) agricultural worker. Spouse's job was coded if higher status than participant. | (1) Professional; (2) trade; (3) skilled labourer; or (4) labourer |
| Birthplace | Question asked to participant: ‘Where were you born?’ Options: (1) city; (2) town; or (3) country. | (1) City; (2) town; (3) country |
| Midlife residence | Question asked to participant: ‘What about between the ages of 20 and 60, where did you live most of the time?’ Options: (1) city; (2) town; or (3) country. | (1) City; (2) town; (3) country |
| Living alone | Question asked to participant: ‘Who lives here?’ Options: with others; (2) alone. | (1) Living alone; (2) living with others |
| Social engagement | All social ties obtained from answers from the participant.   - First social tie: Marital status (married=1) - Second social tie: Living arrangement (not living alone=1) - Third social tie: Participation in social groups (visiting social and/or religious groups=1) - Fourth social tie: Frequency meeting friends (≥1 times per week=1)   Categories: low=0-2 social tie; medium=3 social ties; and, high=4 social ties (in 10/66 Study only able to calculate 0-4 social ties). | (1) High; (2) medium; (3) low |
| Income | Question asked to participant: ‘Do you receive any income, benefits, pensions or allowances?’ Options: (1) no; (2) yes. | (1) Yes; (2) no |
| Smoking status | Questions asked to participant: ‘Has there ever been a period when you smoked cigarettes, cigars, or a pipe, chewing tobacco or snuff nearly every day? and, do you still use tobacco regularly?’ | (1) Never; (2) previous; (3) current smoker |
| Alcohol intake | Question asked to participant: 'What is the most you would drink in a week'? Total drinks per week recorded in standard alcohol units. | (1) No (0 unit per week); (2) light/moderate (1 to 14 standard units per week); or (3) heavy (≥15 units per week) |
| Fish intake | Question asked to participant: ‘How often do you eat fish?’ Options: (1) never; (2) some days; (3) most days; or (4) every day. | (1) Most days/every day; (2) some days; (3) never |
| Fruit and vegetable intake | Question asked to participant: ‘How many servings of fruit and vegetables have you eaten over the last 3 days?’ One fruit or one portion of salad or vegetables counts as a serving. | (1) Daily; (2) not daily |
| Family history of dementia | Question asked to participant: ‘Which relatives father/mother/brother/sister) had this problem?’ Options: (1) not affected; (2) History suggestive of dementia. Those with age of dementia onset <35 year were excluded. | (1) No; (2) yes |
| Physical activity | Question asked to participant: ‘Taking into account both work and leisure, would you say that you are?’ Options: (1) very physically active, (2)  fairly physically active, (3) not very physically active; or (4) not at all physically active. | (1) Very active=0; (2) fairly active; (3) not at all/not very active |
| Difficulty handling money | Question asked to informants: 'Has there been a change in his/her ability to handle money? Options: (1) no difficulty; (2) some difficulty; or (3) cannot handle money | (1) No difficulty; (2) cannot/some difficulty |
| Loneliness | Loneliness was measured using the following question of the LONELINESS section in the Geriatric Mental State Examination AGECAT: ‘Do you feel lonely?’ Responses were: (i) no; (ii) yes, with intensity from mild to moderate; (iii) yes, with intensity severe and frequent; (iv) no reply or not understood; or (v) not asked. | (1) No; (2) yes |
| Weight loss | Weight loss was measured using the following question of the SOMATIC DYSFUNCTION section in the GMS/AGECAT (Q53.1): ‘Have you lost any weight (more than 10lbs) during the past three months?’ Responses were: (i) no; (ii) yes, with intensity from mild to moderate; (iii) yes, with intensity severe and frequent; (iv) no reply or not understood; or (v) not asked. | (1) No; (2) yes |
| Outpatient visit | Question asked to participants: ‘Have you had contact with any primary care/government hospital doctor/other government health workers/private doctor/dentist/traditional healer in the last three months?’ Options: (1) yes; (2) no. | (1) No; (2) yes |
| Hospitalization | Question asked to participants: ‘Have you been admitted to any hospital in the last three months?’ Options: (1) yes; (2) no. | (1) No; (2) yes |
| Self-rated health | Question asked to participant: ‘How do you rate your overall health in the past 30 days?’ Options: (1) very good; (2) good; (3) good; (4) moderate; (5) bad; or (6) very bad. | (1) Very good; (2) good; (3) moderate/bad/very bad |
| Obesity (WHtR) | Two physical measurements were used, height (cm) and WC (cm) as weight was not available. WHtR groupings were generated for males and females as follows: normal (M:< 0.63, F: <0.58); and obese (M: ≥0.63, F: ≥0.58). | (1) Normal; (2) obese |
| Waist circumference | Normal: (M: <94 cm, F: <80 cm)  Increased risk: (M: 94-102 cm, F: 80-88 cm)  High risk: (M: >102 cm, F: >88 cm) | (1) Normal; (2) increased risk; (3) high risk |
| High SBP | The average of two sitting and standing systolic blood pressure measurements. Cut-off value: ≥140mmHg. | (1) No; (2) yes |
| High DBP | The average of two sitting and standing diastolic blood pressure measurements. Cut-off value: ≥90mmHg. | (1) No; (2) yes |
| Hypertension | High SBP and/or DBP | (1) No; (2) yes |
| Sleep problem | Sleep problem was identified using the SOMATIC DYSFUNCTION section of the GMS/AGECAT (Q54.1). Participants were asked if they had trouble with sleep or recent change in pattern. Responses were: (i) no; (ii) yes, with intensity from mild to moderate; (iii) yes, with intensity severe and frequent; (iv) no reply or not understood; or (v) not asked. | (1) No; (2) yes, with intensity from mild to moderate; or (3) yes, with intensity severe and frequent |
| Falls | Participants’ number of falls in last year. | Continuous measure |
| Gait abnormality | Gait abnormality was identified using the GAIT section of the GMS/AGECAT (Q170.4-Q170.9). This section comprises six questions with the following response options: (i) no; (ii) yes, with intensity from mild to moderate; (iii) yes, with intensity severe and frequent; (iv) no reply or not understood; or (v) not asked. Gait abnormality was considered present if at least one positive response was recorded. | (1) No; (2) yes, with intensity from mild to moderate; (3) yes, with intensity severe and frequent |
| Physical frailty | Physical frailty was identified based on the presence of two or more of the following four indicators. (1) Weight loss: self-reported weight loss was assessed using a single item from the GMS/AGECAT (Q53.1). Those reporting weight loss of 10 lbs or more in the last three months were considered to have weight loss. (2) Exhaustion: assessed using a single item (Q.48.1) from the GMS/AGECAT. Participants who reported feeling worn out or exhausted were considered to have this frailty indicator. (3) Slow walking speed: identified based on gait speed. Participants were asked to walk normally to the end of a five-meter length string, turn around, and walk back again. Those who spent 16 seconds or longer were categorized as having slow walking speed. (4) Low energy expenditure: in response to the question ‘Taking into account both work and leisure, would you say that you are: very, fairly, not very, or not at all physically active?’ Those who rated themselves as not at all physically active were considered to have low energy expenditure. | (1) Non-frail; (2) frail |
| Slow physical performance | Identified based on the gait speed. Participants were asked to walk normally to the end of a five-meter length string, turn around, and walk back again. Those who spent 16 seconds or longer were categorized as having slow walking speed. | (1) No; (2) yes |
| Hearing problems | Question asked to participants: ‘How much does hearing difficulty or deafness interfere with your activities; not at all, a little or a lot?’  Responses were: (1) does not have that health problem; (2) has problem, interferes not at all; (3) has problem, interferes a little; or (4) has problem, interferes a lot. Participants with a response of (3) or (4) were regarded as having hearing problems. | (1) No problem; (2) has problem |
| Psychosis | Psychosis was identified based on the GMS/AGECAT. | (1) Non-case; (2) case |
| Difficulties with  ADL/IADL | Questions asked to the informant: ‘Does she/he have difficulty performing household chores that she/he used to do, such as preparing food or boiling a pot of tea?; Has there been a loss of a special skill or hobby she/he could manage before?; Has there been a change in her/his ability to handle money?; Does she/he have difficulty feeding herself/himself?; Does she/he have difficulty dressing?; Does she/he have difficulty using the toilet?; and, Does she/he wet or soil herself/himself? Categories were: no difficulty (no reported difficulty in ADL/IADL); or having difficulty (≥ 1 reported difficulty in ADL/IADL) | (1) No difficulty; (2) having difficulty |
| SMI | Memory impairment was measured using the Identified based on the MEMORY section in the GMS/AGECAT. There were 9 questions (max) with responses (i) no; (ii) yes, with intensity from mild to moderate; (iii) yes, with intensity severe and frequent; (iv) no reply or not understood; or (v) not asked. After excluding the no reply or not asked responses, the sum of the remaining scores was computed and grouped into three categories: (1) No (total score=0); (2) Yes, without worry (total score: 1-3); or (3) Yes, with worry (total score: >3). | (1) No; (2) yes, without worry; (3) yes, with worry. |
| Anxiety | Anxiety was identified based on the GMS‐AGECAT stage 1 anxiety axis. Those having a score of at least three in the GMS/AGECAT Stage I anxiety cluster, which is intended to correspond to clinical levels of anxiety. | (1) No; (2) yes |
| Depression | Depression symptom (diagnosed based on ICD or DSM) | (1) No; (2) yes |
| Total Cholesterol | 15ml blood was collected from the participants for haematology, DNA/genes, and biochemistry analysis. Depending on availability of funding, biochemistry analysis was conducted immediately or within six months. Cut-off value: ≥6.2mm/L. | (1) Not high; (2) high |
| Heart disease | Question asked to participant: ‘Have you ever been told by a doctor that you had heart trouble?’ Options: (1) no; or (2) yes. | (1) No; (2) yes |
| Myocardial infarction | Clinically diagnosed | (1) No; (2) yes |
| Parkinsonism | Clinically diagnosed | (1) Non-case; (2) case |
| Eyesight problem | Question asked to participants: ‘How much does eyesight problem interfere with your activities; not at all, a little or a lot?’ Options:   (1) does not have that health problem; (2) has problem, interferes not at all; (3) has problem, interferes a little; or (4) has problem, interferes a lot. (3) and (4) were regarded as having eyesight problems. | (1) No; (2) yes |
| Thyroid disorder | hypothyroidism | (1) No; (2) yes |
|  | hyperthyroidism | (1) No; (2) yes |
| COPD | Question asked to participants: ‘Have you ever been told that you had COPD?’ | (1) No; (2) yes |
| Arthritis problem | Question asked to participants: ‘How much does arthritis problem interfere with your activities; not at all, a little or a lot?’ Options: (1) does not have that health problem; (2) has problem, interferes not at all; (3) has problem, interferes a little; or (4) has problem, interferes a lot. (3) and (4) were regarded as having arthritis problems. | (1) No; (2) yes |
| Pain | Question asked to participants: ‘In general how would you rate the severity of the pain in the last month?’ Response options were: (1) mild pain; (2) moderate pain; (3) severe pain; (4) very severe pain; or (5) unbearable pain. | (1) No pain; (2) mild/moderate; or (3) severe/very severe/unbearable |
| Diabetes | Question asked to participants: ‘Have you ever been told that you had diabetes?’ | (1) No; (2) yes |
| Verbal fluency | Question asked to participant: ‘I want you to think about all the many different kinds of animals you know. Think of any kinds of animal in the air, on land, in the water, in the forest, all the different animals. Now I would like for you to tell the names of as many different animals as you can. You will have a minute to do this’. Number of animals named in one minute. Participants who named 18 or more animals were regarded as having verbal fluency. | (1) ≥18 animals (2) <18 animals |
| Delayed recall | First, the 10-word list-learning test was completed. Following this, and after a short delay, the participant was asked to recall as many words as possible. Participants were categorized as having recalled 0-4 words, 5-6 words, or 7-10 words. | (1) 7-10 words; (2) 5-6 words; or (3) 0-4 words |
| CDR | The CDR score was based on cognitive and functional information obtained from informants and participants, and a detailed description of operationalisation of this score can be found elsewhere. Categories were: CDR score=0 or CDR score ≥0·5. | (1) CDR score=0; (2) CDR score ≥0·5 |
| Stroke | Clinically diagnosed | (1) No; (2) yes |
| Traumatic brain injury | Question asked to the participant: ‘Have you ever had a serious head injury in which you were knocked out? Options: (1) no; or (2) yes. | (1) No; (2) yes |

Abbreviation: AGECAT, Automatic Geriatric Examination for Computer-Assisted Taxonomy; ADL, activities of daily living; CDR, Clinical Dementia Rating; DBP, diastolic blood pressure; DSM, Diagnostic and Statistical Manual; F, female; GMS, Geriatric Mental State Examination; IADL, instrumental activities of daily living; ICD, International Classification of Diseases; M, male; NA, not applicable; SBP, systolic blood pressure; SMI, subjective memory impairment; TBI, traumatic brain injury; WC, waist circumference; WHtR, waist-to-height ratio.

**Table S3** Description of the LMIC-specific predictors available in the 10/66 Study

| **Predictors** | **Description in 10/66 study** | **Categories** |
| --- | --- | --- |
| Household assets | Question asked to participant with other members of the household present. Numbers of following assets owned in this household: (1) motor vehicles; (2) television; (3) fridge and/ or freezer; (4) water and electricity utilities; (5) telephone; (6) plumbed toilet; and (7) plumbed bathroom. | (1) Higher: upper 50%; (2) lower: lower 50% |
| Food insecurity | Questions asked to participant: ‘Do you ever go hungry because there is not enough food to eat?’ Options: (1) never; (2) some days; (3) most days; or (4) every day. (1) was regarded no food insecurity, while (2), (3), and (4) were regarded as at least occasional food insecurity. | (1) No; (2) yes (at least occasional food insecurity) |
| Late life residence | Question asked to participant: ‘Since you turned 60, where have you lived most of the time?’ Options: (1) city; (2) town; or (3) country. | (1) City; (2) town; (3) country |

**Table S4** Sample size calculation for the survival prediction model.

| **Criterion** | **Required Sample Size** | **Shrinkage** | **Parameters** | **CS-R²** | **EPP** | **Required Events** |
| --- | --- | --- | --- | --- | --- | --- |
| Criterion 1 (shrinkage ≤10%) | 425 | 0.90 | 5 | 0.10 | 8.17 | 41 |
| Criterion 2 (Nagelkerke R² precision) | 198 | 0.808 | 5 | 0.10 | 3.81 | — |
| Criterion 3 (mean risk precision) | 425 | 0.90 | 5 | 0.10 | 8.17 | 41 |
| Final required sample size | 425 | 0.90 | 5 | 0.10 | 8.17 | 41 |

Notes: Calculations were performed using the *pmsampsize* command in Stata. Parameters refer to the number of predictors included in the final model. CS-R² indicates the Cox–Snell R² used as the anticipated model performance. EPP, events per parameter. Required events were estimated using the observed event rate (0.025 per person-year) and mean follow-up time (3.8 years).

**Table S5** Missing data for the selected predictors and LMIC-specific variables across the 10/66 study countries. In the analysis we use case-wise deletion.

| **Predictors** | **Countries** | | | | | | |
| --- | --- | --- | --- | --- | --- | --- | --- |
|  | China | Cuba | DR | Mexico | Peru | Puerto Rico | Venezuela |
| Age, n (%) | None | 6 (0.3) | None | None | None | None | None |
| Social engagement, n (%) | None | None | None | None | None | None | None |
| Physical activity, n (%) | None | 9 (0.4) | 7 (0.5%) | 7 (0.5%) | 6 (0.5%) | 4 (0.3%) | 20 (1.5%) |
| Hypertension, n (%) | 4 (0.2%) | 4 (0.2%) | 11 (0.8%) | 3 (0.2%) | 3 (0.2%) | 181 (13.2%) | 257 (19.0%) |
| Handling money difficulty, n (%) | 6 (0.3%) | 25 (1.1%) | None | 2 (0.1%) | 2 (0.2%) | 1 (0.1%) | 1 (0.1%) |
| Late life residence, n (%) | None | 5 (0.2%) | 2 (0.1%) | None | 5 (0.4%) | 4 (0.3%) | 11 (0.8%) |
| Food insecurity, n (%) | None | 8 (0.3%) | 20 (1.4%) | 9 (0.6%) | 10 (0.8%) | 7 (0.5%) | 56 (4.1%) |
| Household assets, n (%) | None | 3 (0.1%) | None | None | None | None | None |

**Table S6** Point-based risk score (Fine-Gray model) calculations and scoring (based on data from the combined development sample)

| **Risk factor** | **Regression**  **coefficient (β_i_)** | **Reference**  **value (*W*_ij_)** | **βi*(*W*_ij_-*W_i_*_REF_)** | **Points_ij_=**  **βi*(*W*_ij_-*W_i_*_REF_)/*B*** |
| --- | --- | --- | --- | --- |
| Age | 0.0704 |  |  |  |
| 65-69 |  | 67=*W*_1REF_ |  | 0 |
| 70-74 |  | 72 | 0.352 (B) | 1 |
| 75-79 |  | 77 | 0.704 | 2 |
| ≥80 |  | 86 | 1.3376 | 4 |
| Social engagement |  |  |  |  |
| High | Ref. |  |  | 0 |
| Medium | 0.1602 | 1  0=*W*_2REF_ | 0.1602  0 | 0.5 |
| Low | 0.2331 | 1  0=*W*_2REF_ | 0.2331  0 | 1 |
| Physical activity |  |  |  |  |
| Very active | Ref. |  |  | 0 |
| Fairly active | 0.2451 | 1  0=*W*_3REF_ | 0.2451  0 | 0.5 |
| Not at all/not very active | 0.5639 | 1  0=*W*_3REF_ | 0.5639  0 | 1.5 |
| Hypertension |  |  |  |  |
| No | Ref. |  |  | 0 |
| Yes | 0.1118 | 1  0=*W_4_*_REF_ | 0.1118  0 | 0.5 |
| Difficulty in handling money |  |  |  |  |
| No | Ref. |  |  | 0 |
| Yes | 0.7460 | 1  0=*W_5_*_REF_ | 0.7460  0 | 2 |
| Total risk score (0-9) |  | | | |

**Table S7** Point-based risk score from the Fine-Grey model coefficients from each individual country

| **Risk factor** | **Points** | | | | | | |
| --- | --- | --- | --- | --- | --- | --- | --- |
|  | **China** | **Cuba** | **Dominican**  **Republic** | **Mexico** | **Peru** | **Puerto**  **Rico** | **Venezuela** |
| Age |  |  |  |  |  |  |  |
| 65-69 | 0 | 0 | 0 | 0 | 0 | 0 | 0 |
| 70-74 | 1 | 1 | 1 | 1 | 1 | 1 | 1 |
| 75-79 | 2 | 2 | 2 | 2 | 2 | 2 | 2 |
| ≥80 | 4 | 4 | 4 | 4 | 4 | 4 | 4 |
| Social engagement |  |  |  |  |  |  |  |
| High | 0 | 0 | 0 | 0 | 0 | 0 | 0 |
| Medium | 0.5 | 1 | 0 | 0 | 0.5 | 1 | 1 |
| Low | 0.5 | 3 | 0.5 | 0 | 1 | 0 | 0 |
| Physical activity |  |  |  |  |  |  |  |
| Very active | 0 | 0 | 0 | 0 | 0 | 0 | 0 |
| Fairly active | 0.5 | 1 | 0 | 0 | 0 | 2 | 1 |
| Not at all/not very active | 1 | 1 | 0.5 | 1 | 1 | 3.5 | 2 |
| Hypertension |  |  |  |  |  |  |  |
| No | 0 | 0 | 0 | 0 | 0 | 0 | 0 |
| Yes | 0.5 | 1 | 0.5 | 0 | 0 | 0.5 | 0 |
| Difficulty in handling money |  |  |  |  |  |  |  |
| No | 0 | 0 | 0 | 0 | 0 | 0 | 0 |
| Yes | 3 | 2 | 1 | 1.5 | 0 | 2.5 | 2 |
| Total risk score | 0-9 | 0-11 | 0-6.5 | 0-6.5 | 0-6 | 0-11.5 | 0-9 |

**Table S8** Point-based risk score (Fine-Gray model with LMIC-specific predictors) calculations and scoring

| **Risk factor** | **Regression**  **coefficient (β_i_)** | **Reference**  **value (*W*_ij_)** | **βi*(*W*_ij_-*W_i_*_REF_)** | **Points_ij_=**  **βi*(*W*_ij_-*W_i_*_REF_)/*B*** |
| --- | --- | --- | --- | --- |
| Age | 0.0700 |  |  |  |
| 65-69 |  | 67=*W*_1REF_ |  | 0 |
| 70-74 |  | 72 | 0.35 (B) | 1 |
| 75-79 |  | 77 | 0.70 | 2 |
| ≥80 |  | 86 | 1.33 | 4 |
| Social engagement |  |  |  |  |
| High | Ref. |  |  | 0 |
| Medium | 0.1293 | 1  0=*W*_2REF_ | 0.1293  0 | 0.5 |
| Low | 0.2272 | 1  0=*W*_2REF_ | 0.2272  0 | 1 |
| Physical activity |  |  |  |  |
| Very active | Ref. |  |  | 0 |
| Fairly active | 0.2375 | 1  0=*W*_3REF_ | 0.2375  0 | 0.5 |
| Not at all/not very active | 0.5454 | 1  0=*W*_3REF_ | 0.5454  0 | 1.5 |
| Hypertension |  |  |  |  |
| No | Ref. |  |  | 0 |
| Yes | 0.1145 | 1  0=*W_4_*_REF_ | 0.1145  0 | 0.5 |
| Difficulty in handling money |  |  |  |  |
| No | Ref. |  |  | 0 |
| Yes | 0.7516 | 1  0=*W_5_*_REF_ | 0.7516  0 | 2 |
| Late life residence |  |  |  |  |
| City | Ref. |  |  | 0 |
| Town | 0.0972 | 1  0=*W_6_*_REF_ | 0.0972  0 | 0 |
| Country | 0.1195 | 1  0=*W_6_*_REF_ | 0.1195  0 | 0.5 |
| Food insecurity |  |  |  |  |
| No | Ref. |  |  | 0 |
| Yes | -0.0788 | 1  0=*W_7_*_REF_ | -0.0788  0 | 0 |
| Household assets |  |  |  |  |
| Higher | Ref. | 1  0=*W_8_*_REF_ |  | 0 |
| Lower | 0.0008 | 1  0=*W_8_*_REF_ | 0.0008  0 | 0 |
| Total risk score (0-9.5) |  | | | |

**Table S9** Discrimination performance of the risk score-based Fine-Gray model (with and without LMIC-specific predictors) over a mean follow-up of 3.8 years

| **Sample** | **Discrimination** | |  | **Comparison** |
| --- | --- | --- | --- | --- |
|  | Risk score (without  LMIC predictors)  C-statistic (95% CI) | Risk score (with LMIC predictors)  C-statistic (95% CI) |  | (*p*-value) |
| All countries (overall) | 0.74 (0.73, 0.76) | 0.74 (0.73, 0.76) |  | 0.200 |
| Development Sample (overall) | 0.74 (0.72, 0.76) | 0.74 (0.72, 0.76) |  | 0.535 |
| Validation Sample (overall) | 0.75 (0.72, 0.79) | 0.75 (0.72, 0.79) |  | 0.897 |
| China | 0.73 (0.70, 0.77) | 0.73 (0.69, 0.77) |  | 0.403 |
| Cuba | 0.73 (0.69, 0.77) | 0.73 (0.69, 0.77) |  | 0.633 |
| Dominican Republic | 0.71 (0.67, 0.75) | 0.71 (0.67, 0.75) |  | 0.397 |
| Mexico | 0.70 (0.66, 0.75) | 0.70 (0.66, 0.75) |  | 0.399 |
| Peru | 0.82 (0.78, 0.86) | 0.82 (0.78, 0.86) |  | 0.778 |
| Puerto Rico | 0.76 (0.71, 0.80) | 0.76 (0.71, 0.80) |  | 0.221 |
| Venezuela | 0.78 (0.73, 0.83) | 0.78 (0.73, 0.83) |  | 0.226 |

Abbreviations: CI, confidence Interval; LMIC, low- and middle-income country.

**Table S10** Discrimination performance of the risk score-based Fine-Gray model (sex-stratified) at 3-year time horizon

| **Sample** | **Discrimination** | |  | **Comparison** |
| --- | --- | --- | --- | --- |
|  | Male  C-statistic (95% CI) | Female  C-statistic (95% CI) |  | ($p$-value) |
| All countries (overall) | 0.72 (0.69, 0.75) | 0.75 (0.73, 0.77) |  | 0.107 |
| Development Sample (overall) | 0.72 (0.69, 0.75 | 0.75 (0.72, 0.77) |  | 0.239 |
| Validation Sample (overall) | 0.74 (0.68, 0.80) | 0.76 (0.72, 0.80) |  | 0.616 |
| China | 0.74 (0.68, 0.80) | 0.73 (0.68, 0.78) |  | 0.723 |
| Cuba | 0.73 (0.65, 0.80) | 0.73 (0.69, 0.78) |  | 0.874 |
| Dominican Republic | 0.68 (0.60, 0.77) | 0.72 (0.67, 0.77) |  | 0.418 |
| Mexico | 0.71 (0.63, 0.79) | 0.70 (0.65, 0.76) |  | 0.894 |
| Peru | 0.82 (0.78, 0.86) | 0.82 (0.78, 0.86) |  | 0.778 |
| Puerto Rico | 0.72 (0.63, 0.80) | 0.77 (0.72, 0.83) |  | 0.218 |
| Venezuela | 0.74 (0.66, 0.82) | 0.80 (0.75, 0.86) |  | 0.195 |

Abbreviation: CI, confidence interval.

**Table S11** Point-based risk score (excluding China) calculations and scoring

| **Risk factor** | **Regression**  **coefficient (β_i_)** | **Reference**  **value (*W*_ij_)** | **βi*(*W*_ij_-*W_i_*_REF_)** | **Points_ij_=**  **βi*(*W*_ij_-*W_i_*_REF_)/*B*** |
| --- | --- | --- | --- | --- |
| Age | 0.0673 |  |  |  |
| 65-69 |  | 67=*W*_1REF_ |  | 0 |
| 70-74 |  | 72 | 0.3365 (B) | 1 |
| 75-79 |  | 77 | 0.673 | 2 |
| ≥80 |  | 86 | 1.2787 | 4 |
| Social engagement |  |  |  |  |
| High | Ref. |  |  | 0 |
| Medium | 0.1520 | 1  0=*W*_2REF_ | 0.1520  0 | 0.5 |
| Low | 0.1760 | 1  0=*W*_2REF_ | 0.1760  0 | 0.5 |
| Physical activity |  |  |  |  |
| Very active | Ref. |  |  | 0 |
| Fairly active | 0.2385 | 1  0=*W*_3REF_ | 0.2385  0 | 1 |
| Not at all/not very active | 0.5941 | 1  0=*W*_3REF_ | 0.5941  0 | 2 |
| Hypertension |  |  |  |  |
| No | Ref. |  |  | 0 |
| Yes | 0.0920 | 1  0=*W_4_*_REF_ | 0.0920  0 | 0 |
| Difficulty in handling money |  |  |  |  |
| No | Ref. |  |  | 0 |
| Yes | 0.7194 | 1  0=*W_5_*_REF_ | 0.7194  0 | 2 |
| Total risk score (0-8.5) |  | | | |

**Table S12** Discrimination performance of the risk score-based Fine-Gray model (excluding China) at 3-year time horizon

| **Sample** | **Discrimination** | |  | **Comparison** |
| --- | --- | --- | --- | --- |
|  | Risk score  C-statistic (95% CI) | Age alone  C-statistic (95% CI) |  | ($p$-value) |
| All countries (overall) | 0.73 (0.72, 0.76) | 0.71 (0.69, 0.73) |  | <0.001 |
| Development Sample (overall) | 0.74 (0.71, 0.76 | 0.70 (0.68, 0.72) |  | <0.001 |
| Validation Sample (overall) | 0.75 (0.72, 0.78) | 0.73 (0.69, 0.76) |  | 0.014 |
| Cuba | 0.72 (0.68, 0.76) | 0.70 (0.66, 0.74) |  | 0.032 |
| Dominican Republic | 0.70 (0.65, 0.74) | 0.69 (0.65, 0.73) |  | 0.539 |
| Mexico | 0.70 (0.66, 0.75) | 0.69 (0.65, 0.74) |  | 0.352 |
| Peru | 0.82 (0.78, 0.86) | 0.79 (0.74, 0.84) |  | 0.045 |
| Puerto Rico | 0.77 (0.72, 0.81) | 0.72 (0.67, 0.76) |  | <0.001 |
| Venezuela | 0.78 (0.73, 0.82) | 0.72 (0.68, 0.77) |  | 0.001 |

Abbreviation: CI; confidence interval.

Note: Six countries, excluding China, were included in the analysis. The development sample included data from Cuba, Dominican Republic, Puerto Rico, and Venezuela, while the validation sample included data from Mexico and Peru.

**Figure S1** Distribution of the total risk score across the different 10/66 Study sites. Panels display results for: (i) all countries combined and (ii) individual countries (China, Cuba, Dominican Republic, Mexico, Peru, Puerto Rico, and Venezuela).

**Figure S2** Calibration plots of the risk score-based Fine-Gray model by country. Calibration plots show the agreement between predicted 3-year cumulative incidence of dementia from the Fine–Gray risk score and the Locally Estimated Scatterplot Smoothing (LOESS) observed 3-year risks. Panels display results for: (i) all countries combined, (ii) the development sample, (iii) the validation sample, and individual countries (China, Cuba, Dominican Republic, Mexico, Peru, Puerto Rico, and Venezuela). The solid line represents the LOESS-calibrated curve, and the dashed line indicates ideal calibration (perfect agreement between predicted and observed risk). Each panel also reports the calibration slope and intercept with corresponding 95% confidence intervals.

**Figure S3** Decision curve analysis for 3-year dementia risk prediction by country. Net benefit is plotted against threshold probabilities from 0% to 20% for four strategies: the multivariable model (blue solid line), an age-only model (red dashed line), screening all individuals (“treat all”, green dashed line), and screening no individuals (“treat none”, yellow dotted line). Panels display results for: (i) all countries combined, (ii) the development sample, (iii) the validation sample, and individual countries (China, Cuba, Dominican Republic, Mexico, Peru, Puerto Rico, and Venezuela).
